# Tripeptide Leu-Pro-Phe from Corn Protein Hydrolysates Attenuates Hyperglycemia-Induced Neural Tube Defect in Chicken Embryos

**DOI:** 10.1155/2022/4932304

**Published:** 2022-08-29

**Authors:** Chang-Yu Yan, Jie Sun, Gui-Yuan Yu, Jiang-Han-Zi Liu, Rong-Ping Huang, Shao-Cong Han, Qiong-Yi Zhang, Xiao-Min Li, Jian-Gang Yan, Hiroshi Kurihara, Wei-Xi Li, Yi-Fang Li, Rong-Rong He

**Affiliations:** ^1^Guangdong Engineering Research Center of Chinese Medicine & Disease Susceptibility, College of Pharmacy, Jinan University, Guangzhou 510632, China; ^2^Perfect (Guangdong) Co., Ltd., Zhongshan 528451, China; ^3^Yunnan University of Traditional Chinese Medicine, Kunming 650500, China

## Abstract

Neural tube defect (NTD) is the most common and severe embryopathy causing embryonic malformation and even death associated with gestational diabetes mellitus (GDM). Leu-Pro-Phe (LPF) is an antioxidative tripeptide isolated from hydrolysates of corn protein. However, the biological activity of LPF *in vivo* and *in vitro* remains unclear. This study is aimed at investigating the protective effects of tripeptide LPF against NTD in the high glucose exposure condition and delineate the underlying biological mechanism. We found that LPF alleviated NTD in the high glucose-exposed chicken embryo model. In addition, DF-1 chicken embryo fibroblast was loaded with high glucose for induction of oxidative stress and abnormal *O*-GlcNAcylation *in vitro*. LPF significantly decreased accumulation of reactive oxygen species and content of malondialdehyde in DF-1 cells but increased the ratio of reduced glutathione and oxidized glutathione in chick embryo. Oxygen radical absorbance capacity results showed that LPF itself had good free radical scavenging capacity and could enhance antioxidant activity of the cell content. Mechanistic studies suggested that the resistance of LPF to oxidative damage may be related to promotion of NRF2 expression and nuclear translocation. LPF alleviated the overall *O*-GlcNAcylation level of cellular proteins under high glucose conditions and restored the level of Pax3 protein. Collectively, our findings indicate that LPF peptide could act as a nutritional supplement for the protection of development of embryonic neural tube affected by GDM.

## 1. Introduction

In recent years, women during pregnancy have an increased propensity to develop hyperglycemia or gestational diabetes mellitus (GDM). Global estimates revealed that the prevalence of GDM was 16.9% in women aged 20-49 years in 2013 [1]. Newborns and infants of mothers with GDM may have a higher risk of dysplasia or malformation [2]. Neural tube defect (NTD) is the most severe embryopathy with high morbidity, possibly causing anencephaly, microcephaly, exencephaly, and even death. Past studies have shown that development of NTD in embryos exposed to high glucose environment involves multiple cellular mechanisms, including oxidative stress [3], formation of glycation end products [4], expression of microRNAs [5], folic acid metabolism [6], and excessive apoptosis [7].

*O*-linked N-acetyl glucosamine (*O*-GlcNAc) posttranslationally modifies serine and threonine residues of proteins, known as *O*-GlcNAcylation. Emerging evidences have shown that the dysregulation of *O*-GlcNAcylation is associated with the pathogenesis of various diseases, such as diabetes and neurodegenerative diseases [8, 9]. In fact, accumulation of *O*-GlcNAcylation is observed in placenta of hyperglycemic maters and mainly focuses on proteins in endothelial and trophoblast cells [10]. Nuclear factor-E2-related factor 2 (NRF2) is a transcription factor that rapidly responds to oxidative stress. Upon activated, translocation of NRF2 will be performed from the cytoplasm to the nucleus for binding with antioxidant response elements (AREs) and then promoting the transcription of some target genes associated with the cytoprotective defense system against oxidative damage [11]. The NRF2-mediated antioxidant defense system plays a protective role in some diabetic complications, such as diabetic nephropathy and retinopathy [12, 13]. In addition, NRF2 activation could inhibit valproic acid or high glucose-induced NTD in mice [14, 15].

Some plant-derived natural compounds have been utilized to protect high glucose-induced NTD, including epigallocatechin gallate, baicalin, quercetin, and curcumin [16–20]. Our previous study showed that carnosine mitigated high glucose-induced NTD in the chicken embryo model [21]. Recently, the presence of various bioactive peptides has already been reported in many kinds of foods with some health-promoting properties [22]. Corn, an important food crop worldwide, is rich in protein resources that are preserved in corn gluten meal (CGM) after starch is extracted. A variety of active peptides were identified from the hydrolysate and fermentation of CGM [23]. Leu-Pro-Phe (LPF) is a tripeptide isolated from hydrolysate of CGM and shows antioxidative property in some cell-free experiments [24, 25]. However, the biological activity of LPF *in vivo* and *in vitro* remains largely unknown. In addition, numerous studies have shown that redox balance is disrupted under GDM, and antioxidant supplement is an important strategy to protect against embryonic dysplasia. Hence, in this study, we adopted chicken embryo and DF-1 chicken embryo fibroblasts to evaluate the protective effect of LPF peptide against hyperglycemia-induced NTD. Mechanically, we found that LPF activated NRF2-mediated antioxidant response to high glucose-induced oxidative stress besides free radical scavenging. In addition, LPF peptide effectively inhibited protein *O*-GlcNAc modification and then restored the expression of paired box 3 (Pax3), an important transcription factor for neural tube development.

## 2. Materials and Methods

### 2.1. Chemicals and Reagents

LPF peptide was synthesized by Genscript Biotech Corporation (Nanjing, China). D-glucose (G8270) and MTT (M2003) were purchased from Sigma. 2,2′-Azobis(2-methylpropionamidine) dihydrochloride (AAPH, A101386) was from Aladdin Biochemical Technology (Shanghai, China). Sodium fluorescein and 6-hydroxy-2,5,7,8-tetramethylchroman-2-carboxylic acid (Trolox) were from Wako Pure Chemical (Osaka, Japan). Anti-*O*-GlcNAc antibody (CTD110.6) (sc-59623) was from Santa Cruz Biotechnology. Lipid Peroxidation Assay Kit (S0131S), Glutathione (GSH) Assay Kit (S0052), Hematoxylin and Eosin Staining Kit (C0105S), DAPI (C1002), and H_2_DCFDA (S0033S) were purchased from Beyotime Biotechnology (Shanghai, China). Anti-Pax3 antibody was from DSHB. Primary antibodies, anti-NRF2 and anti-Keap1, were purchased from Proteintech. Anti-*β*-Actin and HRP-conjugated secondary anti-rabbit and anti-mouse antibodies were purchased from Fude Biological Technology (Hangzhou, China). Alexa Fluor 488 goat anti-rabbit IgG was purchased from Invitrogen (Carlsbad, CA, USA).

### 2.2. Chicken Embryos and Treatment

Fertilized eggs were incubated in an incubator at 38°C, 65% ~70% relative humidity. High glucose-induced NTD in chick embryos was performed according to our previous study [21]. A window was opened on the blunt side of the egg for the administration of exogenous D-glucose or LPF peptide. Chicken embryos were divided into control group, high-glucose model group, and LPF treatment groups at different concentrations. On embryo development day (EDD) 0, LPF was injected into chicken embryos (2, 10, 50, or 100 nmol/100 *μ*L/egg) from the air cell. On EDD 1, high-glucose model group and LPF treatment groups were stimulated with 0.4 mmol/100 *μ*L/egg D-glucose, while the control group was given the equal volume of bird saline. After treatment, the fertilized eggs were returned to the incubator for further incubation until the required day.

### 2.3. Cell Culture

DF-1 chicken embryo fibroblast was purchased from BeNa Culture Collection (BNCC, Xinyang, China) and cultured in Dulbecco's Modified Eagle's Medium (DMEM) supplemented with 10% fetal bovine serum (FBS) in an incubator at 37°C with 5% CO_2_.

### 2.4. Cell Viability Evaluation by MTT

MTT assay was applied to detect cell viability. DF-1 cells were seeded into a 96-well plate with the density of 4 × 10^3^ cells/well overnight. Cells were treated with indicated concentration of D-glucose for 24 h. After treatment, 10 *μ*L of MTT solution (5 mg/mL) was added to each well for further incubation for 3 h at 37°C. Formazan crystals were dissolved with 200 *μ*L DMSO. The absorbance was determined at 570 nm using a microplate reader.

### 2.5. Determination of Reactive Oxygen Species (ROS) by Flow Cytometry

DF-1 cells were seeded into a 6-well plate with 2 × 10^5^ cells/well overnight. After treatment with LPF for 24 h or not, DF-1 cells were stimulated with D-glucose for 6 h. Subsequently, cells were labeled with 10 *μ*M H_2_DCFDA at 37°C for 20 min and then washed with PBS twice. Cells were harvested by cell scraping and collected by centrifugation at 800 rpm for 5 min. The cell pellet was resuspended in PBS for ROS detection by flow cytometer (Beckman Coulter Epics XL) with FITC fluorescence channel.

### 2.6. Histological Observation

EDD 5 chicken embryos were fixed with 4% paraformaldehyde for 3 days and then embedded in paraffin and sliced into 5 *μ*m sections according to the conventional protocol. Paraffin sections of embryos were dewaxed with xylenes and then rehydrated with an ethanol gradient. According to the instructions of Hematoxylin Eosin (H&E) Staining Kit, sections were stained for 5 min with hematoxylin and washed with flowing water for 10 min. Next, eosin staining was performed for 1 min. All the sections were dehydrated in a gradient ethanol and vitrified by dimethylbenzene according to the conventional protocol. Histological morphology of embryos tissues was observed and photographed under an automatic scanning microscope.

### 2.7. Western Blotting

On EDD 3.5, chicken embryos were taken for Western blotting detection. Embryonic tissue was weighed and added into RIPA lysis buffer at the mass/volume ratio of 1 : 5 with homogenization for total protein extraction. Cell pellets were directly lysed for 30 minutes by RIPA lysis buffer. After centrifugation at 12000 rpm for 4°C, the supernatants were collected, and protein concentrations were quantified by the BCA method. The protein samples were denatured by boiling in 1× loading buffer at 100°C for 10 min. The proteins were separated by SDS-PAGE and then transferred to PVDF membrane. The membrane was blocked with 5% nonfat milk solution at room temperature for 1 h. Blots were incubated with primary antibodies, including anti-Pax3 (1 : 500), anti-NRF2 (1 : 1000), anti-Keap1 (1 : 1000), anti-*O*-GlcNAc (1 : 1000), and *β*-Actin (1 : 3000) at 4°C overnight. Sequentially, HRP-labeled goat anti-rabbit or mouse secondary antibody (1 : 3000) was incubated at room temperature for 2 h. Finally, blots were detected by the ECL substrate system and visualized by a Tanon-5200 Image Analyzer.

### 2.8. Measurement of GSH/GSSG

EDD 5 chicken embryos were homogenized in ice-cold PBS buffer and subsequently centrifuged 4°C for 10 min at 12,000 rpm. The supernatants were subject to measurement of total GSH and reduced GSH by a commercial assay kit according to the manufacturer's instruction: the ratio of GSH and GSSG = reduced GSH/(total GSH − reduced GSH).

### 2.9. Determination of Malondialdehyde (MDA) Content

DF-1 cell lysates were prepared with ultrasound in ice-cold PBS. The supernatants were collected by centrifugation 4°C for 10 min at 12,000 rpm, and the protein level was detected by the BCA method. Determination of MDA content was conducted with a commercial assay kit according to the manufacturer's instruction. The MDA contents were normalized by the protein amount.

### 2.10. Immunofluorescence

DF-1 cells were seeded into 35 mm glass bottom dishes overnight. After indicated treatment, cells were fixed with 4% paraformaldehyde for 5 min followed by being washed in PBS 3 times. Cells were permeabilized with 0.1% Triton X-100 for 15 min and then blocked with 5% bovine serum albumin (BSA) for 1 h at room temperature. Next, cells were incubated with rabbit anti-NRF2 antibody (1 : 100) at 4°C overnight. Alexa Fluor 488 goat anti-rabbit (1 : 500) secondary antibodies were added for 2 h at room temperature in the dark. DAPI was used to label the nucleus for 10 mins at room temperature from light. Fluorescence detection and imaging were conducted with a fluorescence microscope.

### 2.11. Oxygen Radical Absorbance Capacity (ORAC)

ORAC assay applies fluorescence sodium as a fluorescence probe. Trolox acts as a free radical scavenger to protect fluorescence against attack of AAPH-generated peroxyl radical. ORAC reaction was carried out in 75 mmol/L phosphate buffer solution (pH = 7.2). Add LPF or cell lysate sample 20 *μ*L, phosphate buffer 20 *μ*L, and fluorescence sodium 20 *μ*L to a 96-well plate and then add 140 *μ*L AAPH into each well to start the reaction. The microplate was placed in a BioTek microplate reader, and fluorescence intensity was measured every 2 min at the excitation wavelength of 485 nm and the emission wavelength of 527 nm at 37°C. The ORAC value was calculated using the net area under the fluorescence decay curve with Trolox as the standard.

### 2.12. qRT-PCR

Total RNA was isolated from EDD 3.5 chick embryos using TRIzol Reagent (Invitrogen). First-strand cDNA was synthesized using *TransScript* One Step gDNA Removal and cDNA Synthesis SuperMix kit (TransGen Biotech, Beijing, China). Then, PCR amplification of the cDNA was performed with *TransStart* Top Green qPCR SuperMix Kit (TransGen Biotech) on a Roche LightCycler R96 PCR instrument. *β*-Actin gene was used as the housekeeping gene to normalize the target mRNA expression. The relative mRNA expressions were calculated based on the 2^-*ΔΔ*Cq^ method. The primers were synthesized by Sangon Biotech (Shanghai, China). Sequences of the primers used are as follow: *Pax3*: forward CTCAGCGAGAGAGCATCAGC, reverse TTTGTGCGAGTTCTTCCCGA; *Ncam1*: forward TTGTGCTGCTCCTGGTGGCT, reverse TATCGGCGGTGTGCTCTGGTT; *Met*: forward AGCAGTCGGCAGCAGCAGAT, reverse AACTCTCGGCAAGCAGGTCTCC; *Nrf2*: forward CATAGAGCAAGTTTGGGAAGAG, reverse GTTTCAGGGCTCGTGATTGT; *β-Actin*: forward GCTGTGCTGTCCCTGTA, reverse GCTGTAGCCTCTCTCTGTC.

### 2.13. Statistical Analysis

All values are presented as mean ± SD. Statistical significance was analyzed by one-way analysis of variance (ANOVA) followed by Tukey's multiple comparisons test (GraphPad Prism software, San Diego, CA, USA). *p* < 0.05 was considered statistically significant.

## 3. Results

### 3.1. LPF Moderates Hyperglycemia-Induced NTD in EDD 2 Chick Embryos

**Figure 1:**
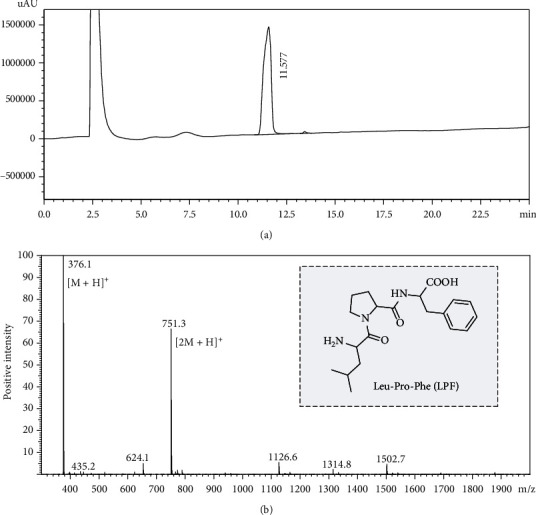
The figure presents HPLC (a) and MS (b) analysis of LPF peptide.

**Figure 2:**
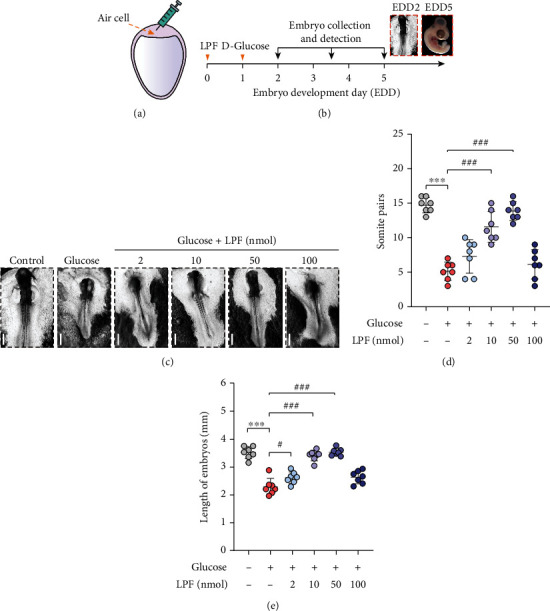
LPF moderates hyperglycemia-induced NTD in EDD 2 chick embryos. (a) Schematic illustration of the administration into chicken embryos by air cell. (b) The time chart illustrating D-glucose or LPF peptide treatment. (c) The representative bright-field images of EDD 2 chicken embryos were taken with a stereomicroscope. The scale bar is 500 *μ*m. (d) Somite pairs and (e) length of EDD 2 chicken embryos were recorded. Data are presented as mean ± SD, and the statistical differences were analyzed by one-way ANOVA. ^∗∗∗^*p* < 0.001, ^#^*p* < 0.05, ^###^*p* < 0.001 vs. the indicated group.

To evaluate the effects of LPF on hyperglycemia-induced NTD in the chick embryo model, sufficient LPF was synthesized and then purified by HPLC followed by MS analysis identification (Figures 1(a) and 1(b)). Chicken embryos gradually begin to close their neural grooves about 25-29 hours after hatching to form the neural tube. Hence, fertilized chick eggs were challenged with high concentration of D-glucose (0.4 mmol/egg) 24 h after hatching (EDD 1) by air cell to produce the NTD model. LPF peptide was injected into chicken embryos at EDD 0 (Figures 2(a) and 2(b)). As shown in Figure 2(c), we observed the morphology of EDD 2 chicken embryos through stereoscopic microscope and found that high glucose exposure caused severe embryonic malformation. In addition, quantitative analysis discovered that high glucose dramatically reduced the somite pair numbers (*p* < 0.001) and length of chick embryos (*p* < 0.001) (Figures 2(d) and 2(e)). However, the addition of 10 nmol/egg or 50 nmol/egg LPF significantly reversed the inhibitory effect of high glucose on embryo development with increased somite pair numbers (*p* < 0.001, *p* < 0.001) and embryonic length (*p* < 0.001, *p* < 0.001) (Figures 2(c)–2(e)). LPF of 100 nmol/egg had no protective effect on hyperglycemia-induced NTD, which suggests that high doses of LPF may have potential adverse effects on embryonic development (Figures 2(c)–2(e)). The above results indicate that LPF could protect against hyperglycemia-induced NTD in EDD 2 chick embryos.

### 3.2. LPF Mitigates Hyperglycemia-Induced NTD in EDD 5 Chick Embryos

**Figure 3:**
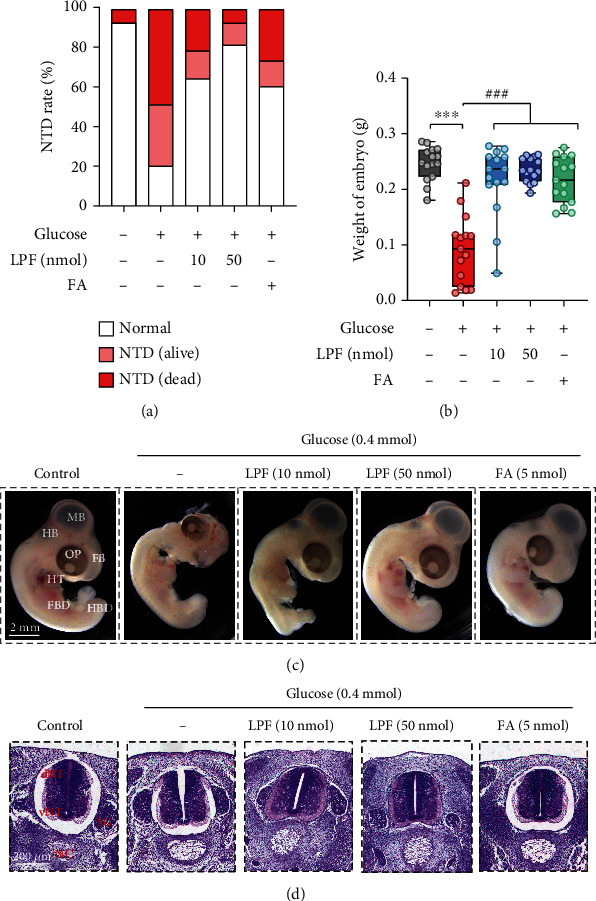
LPF mitigates hyperglycemia-induced NTD in EDD 5 chick embryos. (a) The NTD rate in EDD 5 chicken embryos. (b) The embryonic weight changes in EDD 5 chicken embryos. (c) Morphology of EDD 5 chicken embryos were observed and photographed by a stereomicroscope. Abbreviation: FB: forebrain; MB: midbrain; HB: hindbrain; OP: optic organ; HT: heart; FBD: forelimb bud; HBD: hindlimb bud (HBD). The scale bar is 2 mm. (d) Cross-sections of neural of EDD 5 chicken embryos were stained by the HE method, and pictures were obtained by an automatic scanning microscope. The scale bar is 200 *μ*m. Abbreviation: NC: notochord; SG: spinal ganglion; dNT: dorsal part of the neural tube; vNT: ventral part of the neural tube (vNT). FA (folic acid) was used as a positive drug. Data are presented as mean ± SD, and the statistical differences were analyzed by one-way ANOVA. ^∗∗∗^*p* < 0.001, ^###^*p* < 0.001 vs. the indicated group.

Our previous study found that various patterns of manifestation of NTD caused by high glucose treatment were observed in EDD 5 chicken embryos [21]. Therefore, we next investigated the effect of LPF on hyperglycemia-induced embryonic NTD in EDD 5 chick embryos. As shown in Figure 3(a), high glucose caused obvious NTD and death, while LPF of 10 and 50 nmol/egg lowered the NTD rate and death rate. This indicated that LPF treatment had beneficial effects in improving survival of chicken embryos and preventing the incidence of NTD. Similarly, both doses of LPF treatment significantly reduced the body weight loss of EDD 5 chicken embryos caused by high glucose exposure (*p* < 0.001, *p* < 0.001) (Figure 3(b)). To further assess the protective effect of LPF on hyperglycemia-induced NTD, the morphology of whole mount embryos was observed under a stereomicroscope. As shown in Figure 3(c), the EDD 5 embryos of the control group were structurally complete with good development of all embryonic parts, while high glucose exposure was detrimental to embryonic development and caused malformation. We then observed the neural tubes of the chick embryos by tissue sections and HE staining. Compared with normal embryos with the neural tube well closed, high glucose led to an incomplete closure at the dorsal part of the neural tube (Figure 3(d)). However, both 10 and 50 nmol/egg of LPF improved the morphology of chicken embryos and reduced abnormal closure incidence of neural tubes (Figures 3(c) and 3(d)). Folic acid (FA), a well-known periconceptional agent used as the positive control, also exhibited excellent embryo protection against high glucose-induced NTD (Figures 3(a)–3(d)). The above results indicate that LPF could protect against hyperglycemia-induced NTD in EDD 5 chick embryos.

### 3.3. High Glucose Induces Oxidative Stress and Abnormal *O*-GlcNAcylation in DF-1 Cells

**Figure 4:**
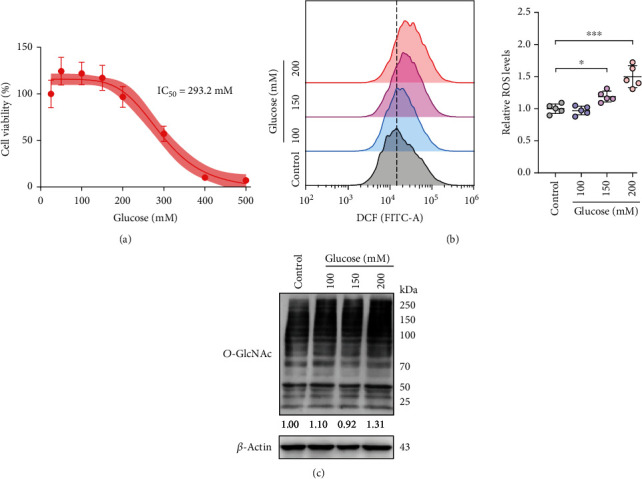
High glucose induces oxidative stress and abnormal *O*-GlcNAcylation in DF-1 cells. (a) The cell viability was examined by MTT assay. DF-1 cells were treated with different concentrations of D-glucose (50-500 mM) for 24 h. (b) DF-1 cells were treated with D-glucose (100, 150, and 200 mM) for 6 h and then stained with H_2_DCFDA for 20 min. The ROS fluorescence signal was measured using a cytometer with FITC channel. The bar graph represents the quantitative analysis of the intracellular ROS level. (c) DF-1 cells were treated with D-glucose (100, 150, and 200 mM) for 24 h. The total *O*-GlcNAcylation level in DF-1 cells was evaluated by Western blotting. The control DF-1 cells were cultured in DMEM medium containing 25 mM D-glucose. Data are presented as mean ± SD, and the statistical differences were analyzed by one-way ANOVA. ^∗^*p* < 0.05, ^∗∗∗^*p* < 0.001 vs. the indicated group.

To further investigate the protective effect of LPF on high glucose exposure induced embryonic dysplasia, DF-1 chicken embryo fibroblasts were applied to construct the cellular model *in vitro*. We first determined the cellular toxicity of D-glucose on DF-1 cells by MTT assay. As shown in Figure 4(a), D-glucose caused death of DF-1 cells with an IC_50_ value of 293.2 mM. Considering that high concentrations of extracellular glucose could lead to cellular water loss and osmotic shrinkage, we used a safe dose of D-glucose within 200 mM in subsequent experiments. H_2_DCFDA is a commonly used probe to detect intracellular ROS. It enters cells freely and is hydrolyzed into DCFH by esterase, which cannot pass through the cell membrane. Intracellular ROS oxidizes nonfluorescent DCFH to fluorescent DCF. Therefore, intracellular levels of ROS can be reflected by detecting fluorescent signals of DCF [26]. We found that D-glucose exposure (150 and 200 mM) significantly increased ROS production in DF-1 cells by flow cytometry analysis (*p* < 0.05, *p* < 0.001), which indicated high glucose-induced oxidative stress *in vitro* (Figure 4(b)). In addition, *O*-GlcNAcylation, a posttranslational *O*-GlcNAc modification on serine and threonine residues of proteins, fluctuates with glucose concentration. Western blotting is widely used to detect levels of intracellular protein *O*-GlcNAcylation [27]. Therefore, we used *O*-GlcNAc-specific antibody to detect the overall levels of protein *O*-GlcNAcylation in cells and found that 200 mM glucose enhanced the levels of cellular *O*-GlcNAcylation (Figure 4(c)). The above results indicate that high glucose induces oxidative stress and abnormal *O*-GlcNAcylation in DF-1 cells.

### 3.4. LPF Inhibits High Glucose-Induced Oxidative Damage in DF-1 Cells and Chicken Embryos

**Figure 5:**
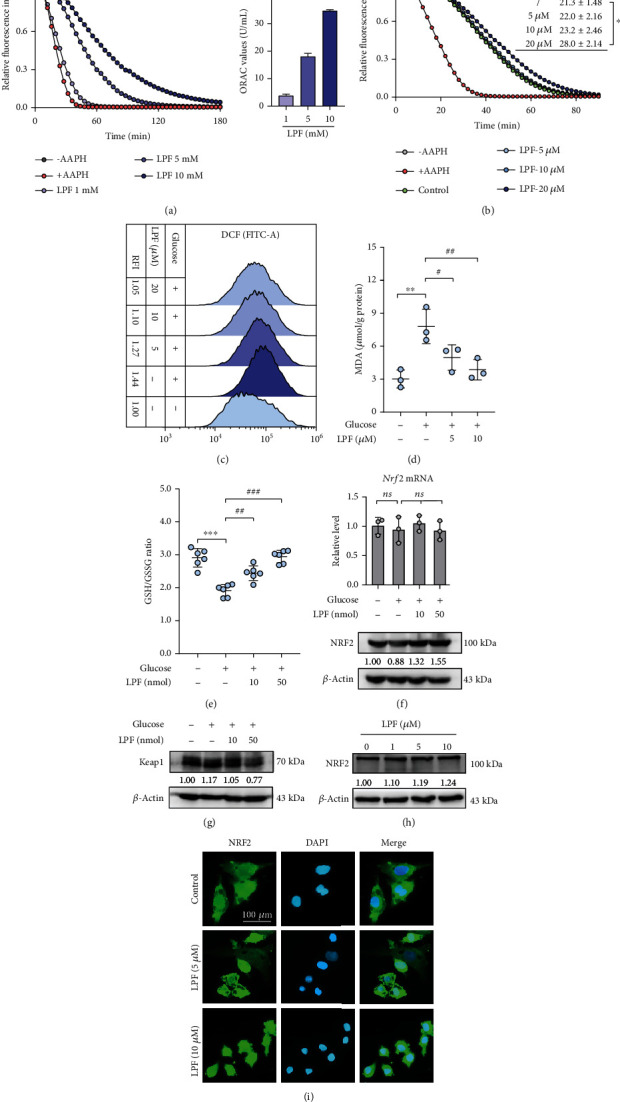
LPF inhibits high glucose-induced oxidative damage in DF-1 cells and chicken embryos. (a) The ROS scavenging capacity of LPF was detected by ORAC in a cell-free assay with AAPH as a ROS generator. The antioxidative activity was calculated as ORAC (U/mL). (b) The ROS scavenging capacity of the DF-1 cell lysates was evaluated by ORAC assay. DF-1 cells were treated with LPF (5, 10, and 20 *μ*M) for 24 h and then lysed with 3 liquid nitrogen freeze-thaw cycles. The supernatants were collected to perform the ORAC experiment. (c) DF-1 cells were treated with different concentrations of LPF for 24 h and then stimulated with D-glucose (200 mM) for additional 6 h. DF-1 cells were stained with H_2_DCFDA probe, and intracellular ROS was measured utilizing a cytometer with FITC channel. The relative fluorescence intensity (RFI) was analyzed quantitatively. (d) DF-1 cells were treated with LPF (5 and 10 *μ*M) for 24 h and then stimulated with D-glucose (200 mM) for additional 24 h. The cells were collected and lysed for detection of MDA content by the assay kit. The MDA contents were normalized by the protein amount. (e) Chick embryos at EDD 5 were homogenized and centrifuged, and the supernatants were collected to measure GSH/GSSG ratio with an assay kit. (f) The mRNA and protein expressions of NRF2 were detected in EDD 3.5 chicken embryo tissues by qPCR and Western blotting. (g) The EDD 3.5 chicken embryonic tissues were lysed, and protein was extracted for Western blotting assay to detect the expressions of Keap1. (h) DF-1 cells were treated with indicated concentrations of LPF for 24 h and then collected for Western blotting detection of NRF2 protein. (i) After stimulated with LPF for 24 h, DF-1 cells were fixed with paraformaldehyde, and NRF2 was detected by immunofluorescence. DAPI was used to label the nucleus for analysis of the nuclear translocation of NRF2. The scale bar is 100 *μ*m. Data are presented as mean ± SD, and the statistical differences were analyzed by one-way ANOVA. ^∗^*p* < 0.05, ^∗∗^*p* < 0.01, ^∗∗∗^*p* < 0.001; ^#^*p* < 0.05, ^##^*p* < 0.01, ^###^*p* < 0.001; ns: not significant vs. the indicated group.

Antioxidant LPF peptide was purified from corn gluten meal hydrolysate, which scavenges a variety of ROS including DPPH radical, ABTS radical, hydroxyl radical, and superoxide radical anion [24, 25]. Therefore, we sought to explore whether LPF exerts antioxidant activity in cells and chick embryo tissue. First, we also evaluated the APPH radical scavenging capacity of LPF by ORAC assay. As shown in Figure 5(a), LPF dramatically protected fluorescein from AAPH radical attack in a concentration-dependent manner, suggesting that LPF directly eliminates ROS. Moreover, the antioxidative activities of LPF-treated DF-1 cells lysates were determined by ORAC assay. LPF (20 *μ*M) significantly enhanced the antioxidative activities of DF-1 cells lysates (*p* < 0.05), suggesting that LPF could enter cells to increase intracellular antioxidant capacity (Figure 5(b)). Based on the above high glucose-induced oxidative stress model in DF-1 cells, we also examined the effect of LPF on intracellular ROS levels using H_2_DCFDA probe. As shown in Figure 5(c), high glucose promoted the production of intracellular ROS, while LPF inhibited the accumulation of ROS dose-dependently. Excessive ROS attacks lipids containing carbon-carbon double bonds such as polyunsaturated fatty acids (PUFAs), commonly known as lipid peroxidation. MDA is one of the metabolism products of lipid peroxidation and is considered a marker of lipid peroxidation [28]. The significantly increased MDA level was found in the high glucose group (*p* < 0.01), while LPF (5 and 10 *μ*M) decreased the content of MDA (*p* < 0.05, *p* < 0.01) (Figure 5(d)). GSH, a nature antioxidant tripeptide with the thiol group, fights various intracellular and extracellular oxidants. GSH is oxidized and regenerated from the oxidized form of GSSG with consumption of NADPH. Hence, GSH/GSSG ratio is considered as an indicator of cellular redox status. Compared with the control group, GSH/GSSG ratio of chicken embryo was significantly decreased in the high glucose group (*p* < 0.001), indicating the oxidative stress response. The treatment of LPF (10 or 50 nmol/egg) significantly increased the GSH/GSSG ratio (*p* < 0.01, *p* < 0.001) (Figure 5(e)). The above results indicate the protective effects of LPF against oxidative stress in DF-1 cells and chicken embryos.

NRF2 plays an important role in antioxidant response to hyperglycemia associated oxidative stress. Activation of NRF2 upregulates the expression of antioxidant genes to promote scavenging of ROS and reduce accumulation of oxidative products [29]. We further investigated whether the antioxidant effect of LPF was associated with activation of the NRF2 signal. First, the expression of NRF2 was detected in EDD 3.5 chicken embryo tissue by qPCR and Western blotting. As shown in Figure 5(f), we observed a downregulation of NRF2 protein level in the high glucose group with the unchanged mRNA expression, which was possibly relevant to the elevated level of Keap1, an endogenous inhibitor of NRF2 (Figure 5(g)). In contrast, LPF treatment inhibited the expression of Keap1 and increased the content of NRF2 protein (Figures 5(f) and 5(g)). Furthermore, we found that LPF promoted the expression of NRF2 in DF-1 cells under normal conditions *in vitro* (Figure 5(h)). Upon activation, NRF2 will translocate into the nucleus and then initiate transcription of downstream genes. Subsequently, we examined the effect of LPF on translocation of NRF2 in DF-1 cells by immunofluorescence assay. The immunofluorescence result showed that LPF (5 and 10 *μ*M) promoted NRF2 translocation from cytoplasm to nucleus (Figure 5(i)). The above results indicate that LPF promotes the expression and activation of NRF2.

### 3.5. LPF Inhibits Hyperglycemia-Induced *O*-GlcNAcylation and Restores Pax3 Protein Level

**Figure 6:**
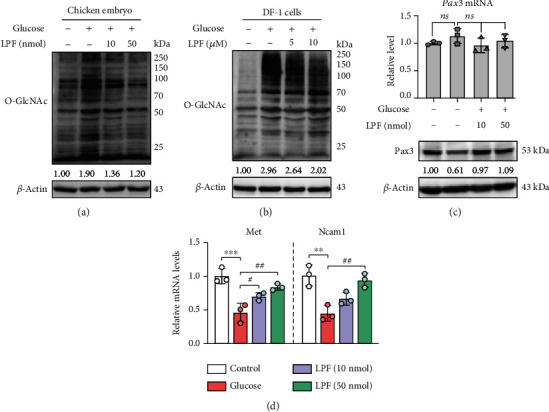
LPF inhibits hyperglycemia-induced *O*-GlcNAcylation and restores Pax3 protein level. (a) The EDD 3.5 chicken embryos were homogenized, and the protein was extracted for SDS-PAGE. The level of *O*-GlcNAcylation was detected by Western blotting using the *O*-GlcNAc antibody. (b) DF-1 cells were treated with LPF (5 and 10 *μ*M) for 24 h and then stimulated with D-glucose (200 mM) for additional 24 h. The cells were collected and lysed for detection of the total *O*-GlcNAcylation level by Western blotting. (c) The mRNA and protein expression of Pax3 was detected in EDD 3.5 chicken embryo tissues by qPCR and Western blotting. (d) The mRNA levels of two downstream genes of Pax3 were detected in EDD 3.5 chicken embryo tissues by qPCR, including *Met* and *Ncam1*. *β*-Actin was set as the housekeeping gene. The expressions of these genes were presented as fold changes relative to the control group. Data are presented as mean ± SD, and the statistical differences were analyzed by one-way ANOVA. ^∗∗^*p* < 0.01, ^∗∗∗^*p* < 0.001; ^#^*p* < 0.05, ^##^*p* < 0.01; ns: not significant vs. the indicated group.

We next investigated the effect of LPF on *O*-GlcNAcylation. As a result, high glucose exposure raised the overall *O*-GlcNAcylation level of EDD 3.5 chicken embryo tissue compared to the control group. Nevertheless, this abnormal *O*-GlcNAcylation of protein was mitigated by LPF treatment (10 and 50 nmol/egg) (Figure 6(a)). Besides, LPF (5 and 10 *μ*M) also alleviated the overall *O*-GlcNAcylation level in high glucose-treated DF-1 cells (Figure 6(b)), suggesting that LPF may regulate the dynamic process of cellular *O*-GlcNAcylation. Pax3, one of the transcription factor Pax family, plays a critical role in neural tube development, and loss or mutation of Pax3 could lead to embryonic NTD [30, 31]. By contrast, restoration of the Pax3 expression in the neural crest contributes to rescuing embryonic development [32]. Our previous study found that high glucose promoted the *O*-GlcNAcylation of Pax3 and led to a decrease in its protein content [21]. Therefore, we detected the effect of LPF on the mRNA and protein levels of Pax3 by qPCR and Western blotting. Consistent with the previous results, high glucose did not significantly affect the transcriptional level of Pax3 (*p* > 0.05), and LPF did not significantly change its mRNA content (*p* > 0.05). Notably, LPF restored the high glucose-induced decrease in Pax3 protein (Figure 6(c)). In order to further confirm the protective role of Pax3 in LPF treatment against high glucose-induced NTD, we detected the downstream gene expression of Pax3 in EDD 3.5 chicken embryo tissues. As shown in Figure 6(d), two Pax3 downstream genes, *Met* and *Ncam1*, significantly decreased under the high-glucose condition (*p* < 0.001, *p* < 0.01), corresponding to a decreased Pax3 protein level in Figure 6(c). LPF treatment significantly increased the mRNA levels of *Met* (10 nmol/egg, *p* < 0.05; 50 nmol/egg, *p* < 0.01) and *Ncam1* (50 nmol/egg, *p* < 0.01) compared to the high-glucose group. The above results indicate that LPF alleviates hyperglycemia-induced *O*-GlcNAcylation and restores Pax3 protein level.

## 4. Discussion

**Figure 7:**
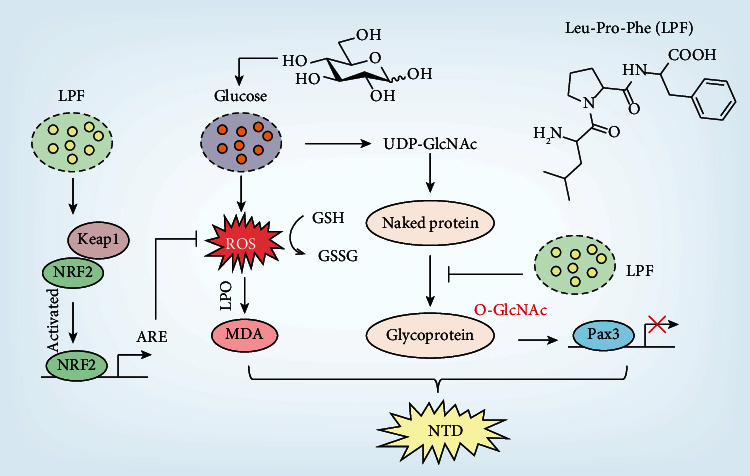
Schematic mechanism of tripeptide LPF alleviating hyperglycemia-induced NTD. LPF ameliorated oxidative stress via activating NRF2 signaling with a decrease in accumulation of ROS and content of MDA and an increase in the ratio of GSH and GSSG. In addition, LPF reduced the abnormal level of *O*-GlcNAcylation level and restored expression of Pax3.

Corn peptide is prepared by enzymatic hydrolysis or fermentation of corn protein, and amino acid sequences of various active peptides have been identified. We found that LPF, derived from corn peptides, alleviated hyperglycemia-induced NTD in the chicken embryos. *In vitro* and *in vivo* results suggest that LPF ameliorated oxidative stress via activating NRF2 signaling, reduced the abnormal level of *O*-GlcNAcylation level, and restored expression of Pax3 (Figure 7). Studies have shown that small peptides could be directly absorbed into the blood circulation through the intestinal barrier. Therefore, biological activities of the oligopeptide can be maintained after oral administration [33]. LPF supplementation is expected to be used to prevent or treat diabetic embryopathy.

The redox balance of the body is maintained by some antioxidant enzymes and reduced molecules in physiological state for protection of tissues and cells from oxidative damage. We found that LPF alleviated high glucose exposure-induced oxidative stress *in vivo* and *in vitro*. Among many endogenous antioxidant molecules, GSH plays the most important role in maintaining redox state. N-Acetylcysteine (NAC), a precursor of cysteine which facilitates GSH supplementation, has been shown to prevent congenital heart defects induced by pregestational diabetes in mice [34]. In addition, NAC could elevate intraplatelet GSH in blood from type 2 diabetes patients and has therapeutic potential for reducing thrombotic risk [35]. Our result shows that LPF peptide increases the ratio of GSH and GSSG in chicken embryo tissues, which may be related to reducing the consumption of GSH by ROS or promoting the recovery of GSH from GSSG. MDA is a reactive aldehyde metabolite of PUFA peroxide and could form adducts with biological molecules such as proteins and nucleic acids. A study found that MDA adduct of hemoglobin maybe associated with significant morbidity in preterm infants [36]. In addition, some clinical studies show that plasma MDA levels in adult type 2 diabetic patients are higher than healthy people [37–39]. Importantly, elevated serum level of MDA is positively correlative of high diabetic peripheral neuropathy score [40]. Our *in vitro* result shows that LPF significantly reduces MDA in high glucose-treated DF-1 cells, possibly in part due to inhibition of ROS by LPF. In fact, many corn peptides, including LPF, have antioxidant activity with free radical scavenging capacity, reducing power and metal chelating activity [23]. LPF shows excellent AAPH free radical (a water-soluble peroxyl radical) scavenging activity in a cell-free system and enhanced the antioxidative ORAC value of LPF-treated DF-1 cells lysates, suggesting that LPF could enter cells to increase intracellular antioxidant capacity.

Dysregulation of redox signaling from the Keap1-NRF2 axis in utero could be an important factor priming disease susceptibility in offspring [41]. Our results also show that Keap1 and NRF2 are affected with marked oxidative stress in the high glucose-induced NTD chick embryo. Accumulating researches have indicated that NRF2 extensively regulates cellular redox status at the transcriptional level. The therapeutic potential of NRF2 activation is widely recognized for oxidative stress and related diseases [42, 43]. Some natural NRF2 activators have been found, including sulforaphane, curcumin, and resveratrol for treatment of hyperglycemia-related diseases [44]. At baseline conditions, NRF2 forms an inactive complex with its endogenous repressor Keap1 on the cytoplasm for degradation by the ubiquitin-proteasome pathway. Therefore, activation of NRF2 could be achieved by reducing the level of Keap1 or interfering with Keap1-NRF2 interaction. Studies have shown that Keap1 degradation can be conducted by autophagic and proteasome-dependent pathway [45, 46]. Notably, some peptide inhibitors have been found to disrupt their interaction based on the structural basis of Keap1 interactions with NRF2 [47–49]. LPF peptide could upregulate the protein expression of NRF2 and promote its translocation from the cytoplasm to the nucleus. Although we found that LPF reduced Keap1 level, the underlying mechanism still needs to be further investigated.

An abnormal *O*-GlcNAcylation modification of the total protein was found in chicken embryos and DF-1 cells with high glucose exposure. Two opposing enzymes, *O*-GlcNAc transferase (OGT) and hydrolase (OGA), dynamically catalyze the install and removal of *O*-GlcNAc, respectively [50]. LPF exerted an inhibitory effect on the overall *O*-GlcNAcylation, which was possibly related to the regulation of both enzymes. In fact, *O*-GlcNAcylation has profound physiological functions in embryonic development. Recent studies have found that *O*-GlcNAcylation modifies some Polycomb group and impacts their functions on regulating early embryogenesis, stem cell differentiation, and other cellular processes [51]. However, excessive *O*-GlcNAcylation has a detrimental effect on embryo development, and OGT inhibition ameliorates NTD in diabetic embryonic mice [52]. Our previous study showed that high glucose led to enhanced *O*-GlcNAcylation modification of Pax3 protein and reduced stability [21]. In addition, the null mutant of Pax3 could cause development of NTD in mice [31, 53]. Hence, upregulation of the Pax3 expression is a potential means to prevent or treat NTD. Although LPF failed to change the mRNA level of Pax3, its protein expression was restored, which was related to the inhibition of LPF on *O*-GlcNAcylation. Pax3 functions as a transcription factor and regulates the expression of multiple downstream genes, such as *Met* and *Ncam1* [54, 55]. Their expressions were decreased after high glucose exposure and recovered by LPF implying restoration of Pax3 function.

## 5. Conclusions

In conclusion, tripeptide LPF was found to alleviate hyperglycemia-induced NTD in chicken embryos and cellular damage in high glucose-exposed DF-1 chicken embryo fibroblasts by regulating oxidative stress and abnormal *O*-GlcNAcylation. Mechanistic studies indicated that the resistance of LPF to oxidative damage may involve promotion of NRF2 expression and nuclear translocation. In addition, LPF could mitigate the overall *O*-GlcNAcylation level of cellular proteins and restore the content of Pax3 protein. Our study demonstrates the protective effect of tripeptide LPF on embryos and promotes its further application as a kind of nutritional supplement for the protection of embryonic neural tube affected by GDM in the future. However, some limitations still exist in the current study. We only focused on investigating the effect of LPF on neural tube at an early stage of chick embryo development. In fact, a high-glucose environment has deleterious effects on the development of various organs and systems. Hence, potential embryo-protected efficacy of LPF needs further evaluation at a later stage of chick embryo development. Moreover, mammalian models like mice have not been utilized for verification of current findings. It is worth noting that mechanistic studies on antioxidation and anti-*O*-GlcNAcylation efficacy of LPF provide further directions for subsequent-related researches. We confirm that antioxidation and anti-*O*-GlcNAcylation are effective therapies to protect embryos against GDM. In addition, supplement of bioactive peptides like LPF is expected to prevent other oxidative stress and *O*-GlcNAcylation-related disease.

## Data Availability

The data used to support the findings of this study are available from the corresponding authors upon request.

## Abbreviations

AREs: Antioxidant response elements
CGM: Corn gluten meal
EDD: Embryo development day
GDM: Gestational diabetes mellitus
GSH: Glutathione
LPF: Leu-Pro-Phe
MDA: Malondialdehyde
NAC: N-Acetylcysteine
NRF2: Nuclear factor-E2-related factor 2
NTD: Neural tube defect
*O*-GlcNAc: *O*-Linked N-acetyl glucosamine
OGA: *O*-GlcNAc hydrolase
OGT: *O*-GlcNAc transferase
ORAC: Oxygen radical absorbance capacity
Pax3: Paired box 3
PUFAs: Polyunsaturated fatty acids.

## Additional Points

*Highlights*. (1) Tripeptide LPF attenuates hyperglycemia-induced neural tube defect. (2) Activation of NRF2 by LPF protects embryos from oxidative damage. (3) LPF restores Pax3 protein level via suppressing hyperglycemia-induced *O*-GlcNAcylation.
